# Retraction: Pro-inflammatory effect of downregulated CD73 expression in EAE astrocytes

**DOI:** 10.3389/fncel.2023.1283223

**Published:** 2023-09-22

**Authors:** Frontiers Editorial Office

The journal retracts the 29 May 2019 article cited above.

Following publication, concerns were raised regarding the integrity of the images in the published figures. Image duplication concerns were identified in Figure 3A. The authors failed to provide a satisfactory explanation during the investigation, which was conducted in accordance with Frontiers' policies. As a result, the data and conclusions of the article have been deemed unreliable and the article has been retracted.

This retraction was approved by the Chief Editor of Frontiers in Cellular Neuroscience and the Chief Executive Editor of Frontiers. The authors did not agree to this retraction.

